# Interaction of Acupuncture and Electroacupuncture on the Pharmacokinetics of Aspirin and the Effect of Brain Blood Flow in Rats

**DOI:** 10.1155/2013/670858

**Published:** 2013-11-25

**Authors:** Ming-Tsang Wu, Lee-Hsin Shaw, Yu-Tse Wu, Tung-Hu Tsai

**Affiliations:** ^1^Institute of Traditional Medicine, School of Medicine, National Yang-Ming University, 155 Li-Nong Street Section 2, Taipei 112, Taiwan; ^2^School of Pharmacy, Kaohsiung Medical University, 100, Shih-Chuan 1st Road, Kaohsiung 80708, Taiwan; ^3^Graduate Institute of Acupuncture Science, China Medical University, No. 91, Hsueh-Shih Road, Taichung 404, Taiwan; ^4^Department of Education and Research, Taipei City Hospital, No. 145, Zhengzhou Road., Datong Dist., Taipei 103, Taiwan

## Abstract

Acupuncture and electroacupuncture have been used to improve the brain and motor functions of poststroke patients, and aspirin is used for the prevention of stroke recurrence. Our hypothesis is that acupuncture and electroacupuncture treatments may interact with aspirin in terms of pharmacokinetics via affecting the brain blood flow. The aim of this study is to investigate the potential interactions of acupuncture and electroacupuncture on the pharmacokinetics of aspirin. The effects of acupuncture treatments on brain blood flow were measured by the laser Doppler blood flow imager. The parallel pharmacokinetic study design included three groups: control, acupuncture, and electroacupuncture groups. Two acupoints, namely, Quchi (LI 11) and Zusanli (ST 36), were needled and stimulated electronically in anaesthetized rats. The concentrations of aspirin and its metabolite, salicylic acid were determined by microdialysis and HPLC analysis after aspirin administration (30 mg/kg, i.v.). The brain blood flow responded to electroacupuncture treatments, but the pharmacokinetic parameters of aspirin and salicylic acid in blood and brain were not significantly changed by acupuncture and electroacupuncture treatments. This study may, in part, offer some evidence to support the contention that there is no significant interaction for the combination of aspirin with acupuncture or electroacupuncture.

## 1. Introduction

Acupuncture, as a part of traditional Chinese medicine, has been used internationally for treatment of many specific diseases, such as hemiplegia and other sequels of brain disease, headache, hypertension, and insomnia according to World Health Organization (WHO) reports [1]. Strokes and their sequelae are another major indication of acupuncture, and early treatment of paresis after stroke has been proved highly effective [2]. Clinically, electroacupuncture has been applied to conventional acupoints such as Baihui (GV20), Yanglingquan (GB34), Hegu (LI4), Quchi (LI 11), and Zusanli (ST 36) for poststroke patient rehabilitation [3, 4]. Two acupoints, Quchi (LI 11) and Zusanli (ST 36), have been suggested to have potential benefits for post-stroke conditions such as spastic paralysis [5] and improved stroke patients' symptoms and signs [6]. Experimental animal data also support that electro-acupuncture treatment at Quchi (LI 11) and Zusanli (ST 36) have neuroprotective function [7] and cerebral protective function [8]. Acupuncture and electro-acupuncture have clearly shown benefits for post-stroke patients [9, 10]. It is suggested that acupuncture can increase blood flow of the peripheral, mesenteric, and retrobulbar arteries [11, 12]. This blood flow is an important supporting factor to drug absorption, distribution, metabolism, and excretion, so if the blood flow changes, the pharmacokinetic parameters of drug might be affected. 

Aspirin, an acetylated salicylic acid, is classified as one of the nonsteroidal anti-inflammatory drugs (NSAIDs). Aspirin is the most commonly used drug for relieving pain, inflammatory symptoms, and fever. It also has established efficacy for preventing myocardial infarction and ischemic stroke, as well as for treating acute myocardial infarction [13]. In a previous study, low-dose aspirin has been shown useful for the prevention of cardiovascular diseases and stroke recurrence [14]. Furthermore, low-dose aspirin can also reduce mortality rates of cardiovascular disease and stroke patients [15]. For post-stroke patients, it may be beneficial to undergo treatment by electro-acupuncture combined with aspirin [16]. But adverse reactions, of which there is a wide spectrum, frequently accompany anti-inflammatory doses of aspirin. Aspirin and related derivatives of salicylic acid have been reported to have a wide range of drug interactions but relatively few seem to be clinically important. Many of these interactions are pharmacokinetic in nature [17]. Cyclooxygenase is irreversible inhibition by aspirin, and dose-related side effects of aspirin on gastrointestinal symptoms (e.g., bleeding complications) have been reported [18, 19]. In addition, aspirin exhibits a nonlinear pharmacokinetics, meaning that the binding of drugs to plasma components, blood cells, and extravascular tissue may demonstrate concentration dependence. Therefore, if acupuncture changes the pharmacokinetics of aspirin, the side effects may occur.

A survey from PubMed with the key words of electro-acupuncture and pharmacokinetics of aspirin, found no items. Accordingly, this study develops a technique using microdialysis coupled to liquid chromatography with photodiode-array detection to monitor protein-unbound aspirin and its metabolites in blood and brain of the rat. To investigate potential interactions between acupuncture and electro-acupuncture on the pharmacokinetics of aspirin, microdialystates of the blood and brain on the groups of control, acupuncture, and electro-acupuncture were individually collected and measured to explore the pharmacokinetic interaction of aspirin. To monitor the rat brain blood flow, a laser Doppler blood flow imager was used to measure the brain blood flow on the groups of control, acupuncture and electro-acupuncture. The study may, in part, provide important information regarding the effects of acupuncture and electro-acupuncture as they are influenced by the pharmacokinetic and pharmacodynamic interaction of aspirin. Physiological factors affecting the distribution of a drug include plasma protein binding, blood perfusion, and membrane permeability. Previous papers have discovered that acupuncture changes blood flow [12, 20], which may affect directly the distribution of a drug. In this work, we assessed the effects of acupuncture on blood flow to confirm the reported results and then performed pharmacokinetic experiments. We decided to use a rat model by microdialysis sampling to examine the effects of acupuncture/electro-acupuncture on drug distribution, because microdialysis provides several advantages such as sampling protein-unbound drug, providing high temporal resolution data and minimizing physiological disturbance of the subject, for a pharmacokinetic evaluation [21]. Aspirin had poor oral bioavailability, which may lead to fluctuated pharmacological effects [22], and we chose intravenous route for drug administration to minimize the fluctuated pharmacological effects caused by inter-subject absorption variations. The aim of our work is to evaluate the possible interactions between acupuncture and pharmacokinetics of aspirin. The use of acupuncture and electro-acupuncture may result in various biological effects on the subject, so we attempted to find the relationship between acupuncture and pharmacokinetics from a distribution viewpoint.

## 2. Materials and Methods

### 2.1. Chemicals and Reagents

Aspirin, salicylic acid, *α*-chloralose, and urethane were purchased from Sigma-Aldrich Chemicals (St. Louis, MO, USA). Acetic acid, sodium citrate, dextrose, sodium chloride, potassium dihydrogen phosphate (KH_2_PO_4_), and orthophosphoric acid (85%, w/w) were purchased from E. Merck (Darmstadt, Germany). Acetonitrile of analytical grade was purchased from ECHO Chemical Co. (Taiwan). Deionized water from Millipore (Milford, MA, USA) was used for all aqueous solutions in this study. 

### 2.2. HPLC Instrumentation

HPLC-UV instrumentation consisted of a Shimadzu chromatographic pump (LC-20AT), a DGU-20A5 degasser, an autosampler (SIL-20AC) and a photodiode array detector (SPD-M20A) (Shimadzu, Kyoto, Japan). A reverse-phase C18 column (Merck, Purospher STAR, 250 mm × 4 mm i.d.; particle size 5 *μ*m, Darmstadt, Germany) was used to separate the analytes. The UV absorbance wavelength was set at 240 nm to detect aspirin and salicylic acid. The mobile phase consisted of acetonitrile 10 mM KH_2_PO_4_ (29 : 71, v/v, pH 2.5 adjusted by orthophosphoric acid) for analysis of blood and brain microdialysates. A Millipore (0.22 *μ*m) filter (Bedford, MA, USA) was used to filter the mobile phase and we used a sonicator (Branson, CT, USA) to degas before the mobile phase was used. The flow rate was set at 1 mL/min.

### 2.3. Experimental Animals

Adult, male, pathogen-free Sprague-Dawley rats (200–260 g) were obtained from the Laboratory Animal Center at National Yang-Ming University (Taipei, Taiwan). Rats were housed in cages with 12 h light/dark cycle; food (Laboratory Rodent Diet 5001, PMI Feeds Inc., Richmond, IN, USA) and water were available *ad libitum*. The animals were received at 6-7 weeks of age and acclimated for at least one week. All animal experiments followed the National Yang-Ming University guidelines and procedures for the care of laboratory animals and the protocol listed above has been reviewed and approved by the Institutional Animal Care and Use Committee (IACUC; approval number 1011203) by the Institutional Animal Experimentation Committee of National Yang-Ming University. The animals had free access to food and water. The rat was anaesthetized with urethane 1.0 g/mL and *α*-chloralose 0.1 g/mL (1 mL/kg, i.p.) before surgery. The femoral vein was cannulated for further drug administration, and the rat's body temperature was maintained by a heating pad during the experiment and they were euthanized by overdose CO_2_ under the anesthetic after the experimental endpoint.

### 2.4. Microdialysis Experiments

The microdialysis system included a CMA/100 microinjection syringe pump, a CMA/140 microfraction collector (CMA, Stockholm, Sweden), and corresponding microdialysis probes located at sampling sites. The microdialysis probes for blood and brain sampling were made in our laboratory. Briefly, the dialysis membranes (150 *μ*m outer diameter with a nominal molecular weight cutoff of 13,000; Spectrum Co., Laguna Hills, CA, USA) for blood and brain are 10 mm and 3 mm in length, respectively [23]. All unions were cemented with epoxy adhesive, and the probes were made at least 24 h prior to use to allow for adequate time for the epoxy adhesive to harden. The blood microdialysis probe was located within the jugular vein/right atrium and perfused with an anticoagulant dextrose solution (ACD, citric acid 3.5 mM; sodium citrate 7.5 mM; dextrose 13.6 mM). The brain microdialysis probe was implanted in the right striatum (coordinates AP −0.2 mm, ML −3.0 mm, DV −7.5 mm) and perfused with Ringer's solution. The flow rates of ACD and Ringer's solution were set at 2.0 *μ*L/min by a microinjection syringe pump for blood and brain microdialysis. The implantation positions of the probes were verified by standard histological procedure at the end of experiments. After different pretreatment in three groups, the dialysates were collected every 15 minutes for 6 hours and preserved under −20°C refrigeration. A validated HPLC-UV system was applied to determine the concentration of aspirin and salicylic acid in the dialysates of blood and brain. A retrodialysis (*R*_dial_) method was used to estimate in vivo recovery, following the method in our previous report [24].

### 2.5. Acupuncture and Electro-Acupuncture for Pharmacokinetic Study

The study design was divided into the three groups of control, acupuncture, and electro-acupuncture groups. Each group included six SD rats (*N* = 6). In the control group, aspirin was administered alone (30 mg/kg, i.v.) injected via the femoral vein. The acupuncture and electro-acupuncture groups were, respectively, treated with acupuncture and electro-acupuncture for 15 minutes and then aspirin was administered (30 mg/kg, i.v.) via the femoral vein, respectively.

After the microdialysis experimental rat model was set up, the stainless steel acupuncture needles (outer diameter of 0.28 mm) were sterilized with 75% alcohol before treatment. Then the needles were inserted into the rat at bilateral acupoints corresponding to the Quchi acupuncture point (LI 11) and the Zusanli acupuncture point (ST 36) in humans [25]. In the acupuncture group, the acupuncture needles remained inserted for 15 minutes before aspirin was injected via the femoral vein. In the electro-acupuncture group, the acupuncture needles were connected to an electrotherapeutic apparatus (Model-058, Ching-Ming, Taiwan) and treatment continued for 15 minutes before aspirin was injected via femoral vein. The electro-acupuncture parameters were set as a disperse-dense wave, frequency of 2 and 50 Hz, and an intensity of 1 mA [26].

### 2.6. Measurement of Brain Blood Flow

The skull of the anaesthetized was exposed and a laser Doppler blood flow imager (moorLDI2, Moor Instruments, UK) was used to monitor the rat brain blood flow. To obtain the baseline control data, the rat brain blood flow was measured before acupuncture or electro-acupuncture stimulation. Then the rat brain blood flow was continually measured during acupuncture or electro-acupuncture stimulation for 6 hours. The rat blood flow data was analyzed and calculated by MoorLDI Version 5 Research Software. 

### 2.7. Pharmacokinetic Data Calculation

The concentrations of aspirin and salicylic acid in the dialysate (*C*_*m*_) were converted to protein-unbound concentrations (*C*_*u*_) by the following equation: *C*_*u*_ = *C*_*m*_/*R*_dial_. Each individual set of data was used to calculate the pharmacokinetic parameters by the pharmacokinetic program, WinNonlin Standard Edition Version 1.1 (Scientific Consulting, Apex, NC, USA). Pharmacokinetic parameters of elimination half-life (*t*_1/2_), area under the concentration-time curve (AUC), clearance (Cl), and apparent volume of distribution (*V*_*d*_) were used in this study.

### 2.8. Statistical Analysis

All data are presented as mean ± standard error of mean (S.E.M.). One-way ANOVA and post hoc analysis were carried out for statistical comparison between the control, acupuncture, and electro-acupuncture group using the statistical software. The version of SPSS is 10.07 (SPSS, Chicago, USA), and the *P* < 0.05 was considered statistical significantly.

## 3. Results

### 3.1. Analytical Method

**Figure 1:**
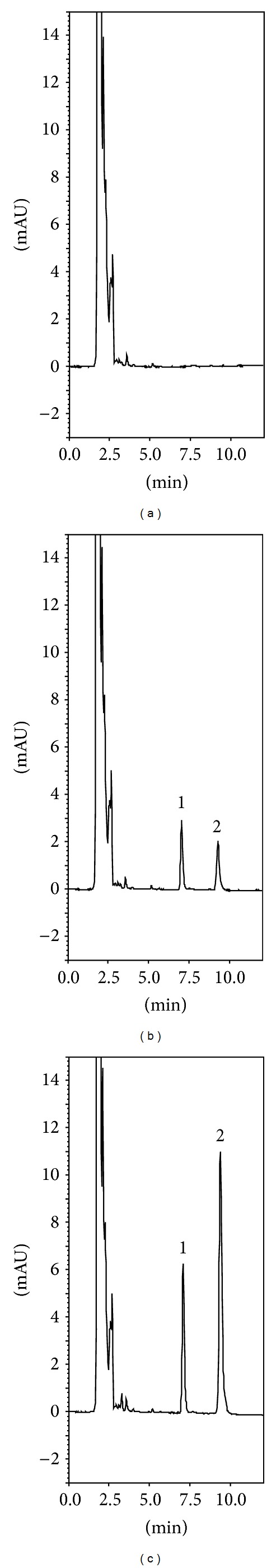
Typical chromatograms of (a) blank blood dialysate; (b) blank blood dialysate spiked with ASA (1 *μ*g/mL) and SA (1 *μ*g/mL); (c) blood sample containing ASA (2.1 *μ*g/mL) and SA (5.6 *μ*g/mL) after administration of aspirin (30 mg/kg, iv) at 30–45 min. (1) Acetylsalicylic acid (ASA); (2) salicylic acid (SA).

**Figure 2:**
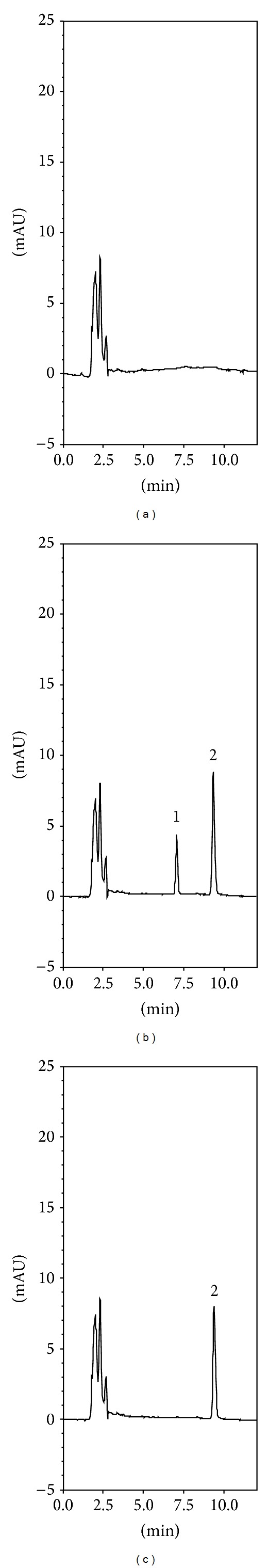
Typical chromatograms of (a) blank brain dialysate; (b) blank brain dialysate spiked with ASA (1.5 *μ*g/mL) and SA (1.5 *μ*g/mL) and; (c) brain sample containing SA (1.3 *μ*g/mL) collected at 105–120 min after administration of aspirin (30 mg/kg, i.v.). (1) Acetylsalicylic acid (ASA); (2) salicylic acid (SA).

Aspirin and salicylic acid were separated by acidic mobile phases which were adjusted to acidity with 10 mM KH_2_PO_4_/acetonitrile (71 : 29, v/v, pH 2.5 adjusted by orthophosphoric acid) [24]. Typical chromatograms of aspirin and salicylic acid in rat blood and brain dialysates are shown in Figures 1 and 2. The retention times of aspirin and salicylic acid were 7 and 9.8 min, respectively.

The chromatogram of a blank blood dialysate is shown in Figure 1(a). The chromatogram of standard aspirin (1 *μ*g/mL) and salicylic acid (1 *μ*g/mL) is shown in Figure 1(b). The real blood samples containing aspirin (2.1 *μ*g/mL) and salicylic acid (5.6 *μ*g/mL) were collected at 30–45 min dialysate after aspirin administration (30 mg/kg, i.v.).  Figure 2(a) shows the chromatogram of a blank brain dialysate.  Figure 2(b) shows the chromatogram of standard aspirin (1.5 *μ*g/mL) and salicylic acid (1.5 *μ*g/mL).  Figure 2(c) shows the real brain dialysate containing only salicylic acid (1.3 *μ*g/mL) collected at 105–120 min after aspirin administration (30 mg/kg, i.v.). 

### 3.2. Pharmacokinetics of Aspirin and Salicylic Acid in Blood and Brain

**Figure 3:**
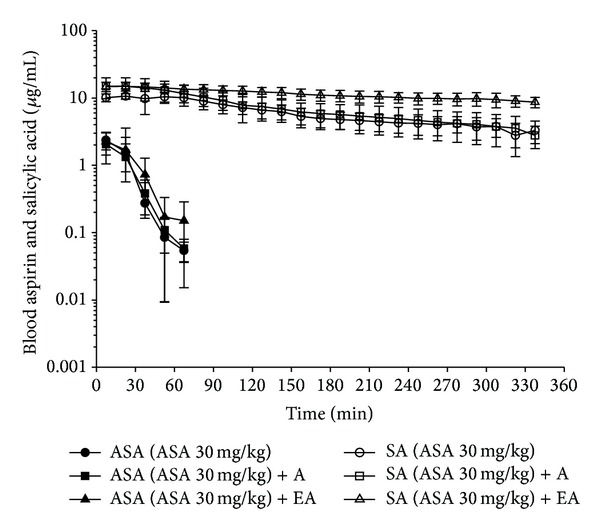
Concentration-time curve of protein-unbound aspirin and salicylic acid in the blood after aspirin administration at 30 mg/kg. The control group (aspirin administration only): concentration of ASA (●), concentration of SA (○). The acupuncture group (acupuncture stimulation 15 min before aspirin administration): concentration of ASA (■), concentration of SA (□). The electro-acupuncture group (electro-acupuncture stimulation 15 min before aspirin administration): concentration of ASA (▲), concentration of SA (∆). Acetylsalicylic acid (ASA); salicylic acid (SA). Acupuncture (A); electro-acupuncture (EA).

**Figure 4:**
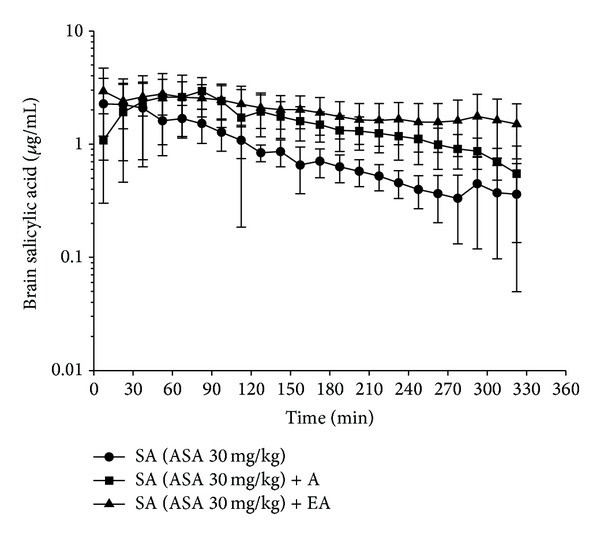
Concentration-time curve of protein-unbound salicylic acid in the brain after aspirin administration 30 mg/kg. The control group (aspirin administration only): concentration of SA (●). The acupuncture group (acupuncture stimulation 15 min before aspirin administration): concentration of SA (■). The electro-acupuncture group (electro-acupuncture stimulation 15 min before aspirin administration): concentration of SA (▲). Salicylic acid (SA). Acupuncture (A); electro-acupuncture (EA).

Figures 3 and 4 show the concentration-time curves of aspirin and salicylic acid in blood and brain, respectively, for the groups of control, acupuncture, and electro-acupuncture after aspirin administration (30 mg/kg, i.v.). Tables 1 and 2 show the pharmacokinetic parameters of aspirin and salicylic acid in the control, acupuncture and electro-acupuncture group in rat blood and brain, respectively. However, aspirin could not be observed in the brain at the dosage of aspirin administration (30 mg/kg). The in vivo hydrolysis of aspirin occurs very rapidly in human, which makes clinicians dependent majorly on the determination of salicylate to assess the therapeutic progress [27]. In our study, aspirin can be only observed in the blood microdialysis samples, and salicylic acid can be detected in the blood and brain microdialysis samples (Figures 3 and 4). The pharmacokinetic data (Tables 1 and 2) demonstrate that acupuncture, and electro-acupuncture did not significantly interact with the concentration of aspirin and salicylic acid in the blood and brain after aspirin administration (30 mg/kg, i.v.).

### 3.3. Rat Brain Blood Flow

**Figure 5:**
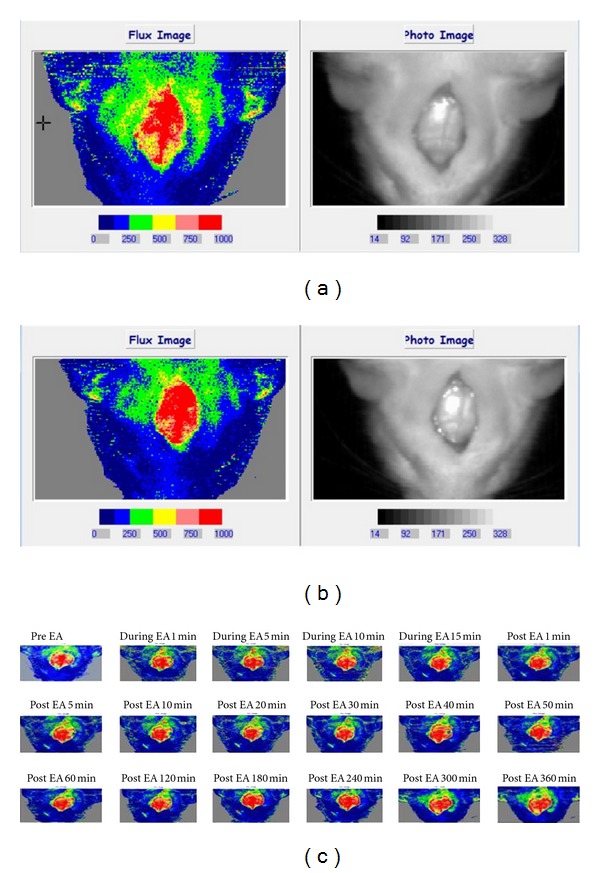
Rat brain blood flow image. (a) Before electro-acupuncture stimulation; (b) after electro-acupuncture stimulation for 5 min; (c) continuous monitoring for 6 h.

**Figure 6:**
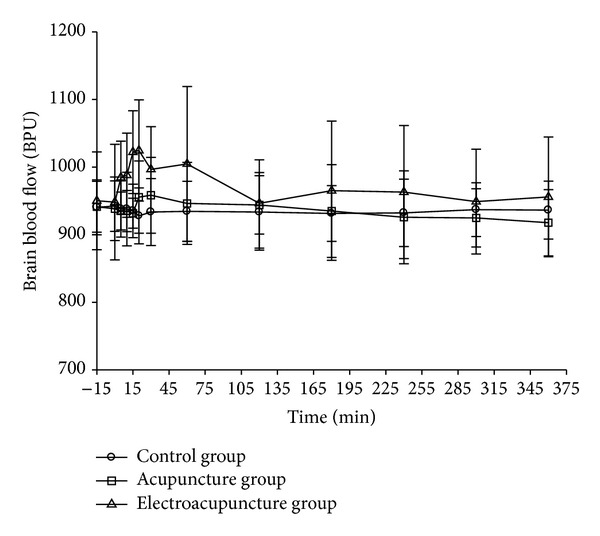
Rat brain blood flow: control group (●); acupuncture group (■); electro-acupuncture group (▲); EA: electro-acupuncture group.

Figure 5 shows the rat brain blood flow images. In Figures 5(a) and 5(b), increased blood flow was found after electro-acupuncture stimulation for 5 min.  Figure 5(c) shows the brain blood flow for consecutive 6 h after electro-acupuncture stimulation.  Figure 6 and Table 3 demonstrated the integrated brain blood flow of control, acupuncture and electro-acupuncture groups. A 15-minute period before treatment (−15–0 min) was used as the baseline for brain blood flow measurement. Then during the time of 0–15 min there was treatment with different stimulation for the acupuncture and electro-acupuncture groups. After stimulation (15 min), the needles were removed. The data demonstrate that the brain blood flow increased in the electro-acupuncture group more than in the groups of control and acupuncture. The time to reach the highest brain blood flow is around 15 min after electro-acupuncture stimulation.

## 4. Discussion

According to the formula for dose translation based on the body surface area [28], the human equivalent dose is 4.86 mg/kg, which equals approximately 291 mg aspirin for a 60 kg adult. The dose (30–325 mg) of aspirin has been used for the secondary prevention of vascular events after ischaemic stroke [29]. Patients with recent symptomatic lacunar infarcts identified by magnetic resonance imaging have received 325 mg of aspirin daily to evaluate the reduction of the risk of recurrent stroke and the risk of bleeding and death [30]. The dose selection for aspirin is acceptable and reasonable.

The absorption, distribution, metabolism, and elimination of a drug are influenced by various physiological factors, such as plasma protein binding, blood perfusion, membrane permeability, enzymatic metabolism, and membrane transports [31]. In our current study, we only focus on the effects of altered blood perfusion caused by electro-acupuncture on the pharmacokinetics of aspirin. However, electro-acupuncture may have additional biological effects on the subject. The effects of electro-acupuncture on metabolic enzymes, transporter activities, and plasma protein expression have to be evaluated in the future studies. 

Urethane and *α*-chloralose were used as anesthesia agents in this study. Anaesthesia using urethane-chloralose is a commonly used combination for pharmacokinetic studies and has been considered acceptable for pharmacokinetic-pharmacodynamic studies [32]. Our study included the control group to exclude the effects caused by interactions between anesthesia agents and aspirin. Although the combination of acupuncture, electro-acupuncture, herbal medicine and Western drug therapy has been used in clinical applications for several decades, there has been little research on the interaction between acupuncture or electro-acupuncture and the pharmacokinetics of Western drugs. In 2004, we developed a microdialysis system to explore the interaction of acupuncture on the acupoints of Taichong (LR3) and Yanglingquan (GB34) for the pharmacokinetics of geniposide in rats [33]. The results indicated that these two acupoints did not affect the pharmacokinetics of geniposide in rat blood, liver, and bile in that experimental model. However, Zhou et al. (2009) showed that electro-acupuncture at the acupoints of Jizhong (GV6), Dazhui (GV14), and Zhongwan (CV12) increases the absorption of baicalin from extracts of *Scutellaria baicalensis* Georgi in normal rats [25]. However, based on the previous results, there is no consensus for the effects of acupuncture or electro-acupuncture on the pharmacokinetics of herbal medicine. In this study, the pharmacokinetic data demonstrate that stimulation with acupuncture and electro-acupuncture had little significant effect on the concentration of aspirin and salicylic acid in the blood and brain after aspirin administration (30 mg/kg, i.v.). One potential explanation is that several factors may affect these results, such as the anesthetized experimental animal model, the acupoints selected, the dose of aspirin, or the protein-unbound form of analytes collected by microdialysis. Furthermore, acupuncture or electro-acupuncture stimulation of physiological functions, for example, the autonomic nervous system or organ blood flow, can indirectly affect the absorption, distribution, metabolism, and excretion of drugs [34].

Another possible explanation is that the short-term and long-term stimulation of acupuncture and electro-acupuncture may have different results. In our study, we try to confirm the short-term acupuncture and electro-acupuncture stimulation interaction on the pharmacokinetics of aspirin in rats. However, long-term stimulation of acupuncture and electro-acupuncture points would be a very interesting subject for future studies. 

Recent studies have found that the mechanism of acupuncture is partly related to the nervous and vascular system [35]. Thus, the effect of acupuncture and electro-acupuncture stimulation in awake or anesthetized subjects might be different. After having been under anesthesia for a period of time, a subject's physiological responses to acupuncture or electro-acupuncture might be concealed. To perform acupuncture and electro-acupuncture stimulation on laboratory animals and for experimental feasibility considerations, an anesthetized animal model for blood and brain sampling was used. To avoid the anesthesia effect on acupuncture, a well-designed clinical trial should be performed.

## 5. Conclusions

In this study, our data demonstrate that acupuncture and electro-acupuncture did not significantly interact with the pharmacokinetics of aspirin (30 mg/kg, i.v.) in rat blood and brain. According to our results in this work, the use of acupuncture and electro-acupuncture did not change the distribution and pharmacokinetics of single-dose aspirin in rats, which might suggest in part the safety of combination of acupuncture and electro-acupuncture and aspirin. However, further studies are necessary to clarify other potential mechanisms of acupuncture and electro-acupuncture that influence pharmacokinetics. 

## Figures and Tables

**Table 1:** The pharmacokinetic parameters of aspirin and salicylic acid for the rat blood. The control group (aspirin administration 30 mg/kg only); the acupuncture group (acupuncture stimulation 15 min before aspirin administration 30 mg/kg); the electro-acupuncture group (electro-acupuncture stimulation 15 min before aspirin administration 30 mg/kg).

Parameter	Aspirin (30 mg/kg)	Aspirin (30 mg/kg) + acupuncture	Aspirin (30 mg/kg) + electro-acupuncture
Aspirin			
*t*_1/2_ (min)	12 ± 2	11 ± 2	12 ± 4
AUC (min *μ*g/mL)	113 ± 33	95 ± 10	92 ± 5
Cl (mL/min/kg)	291.8 ± 62.4	333.0 ± 70.5	343.0 ± 50.1
Vd (mL/kg)	3.6 ± 0.8	3.8 ± 1.9	3.9 ± 0.5
Salicylic acid			
*t*_1/2_ (min)	289 ± 46	215 ± 22	225 ± 15
AUC (min *μ*g/mL)	2854 ± 518	2565 ± 322	2304 ± 220
Cl (mL/min/kg)	8.6 ± 2.6	9.3 ± 1.1	10.4 ± 1.5
Vd (mL/kg)	3.3 ± 1.0	2.5 ± 0.6	2.65 ± 0.1

Data expressed as mean ± S.E.M. (*n* = 6). *t*_1/2_: elimination half-life, AUC: area under the concentration-time curve, Cl: clearance, Vd: apparent volume of distribution.

**Table 2:** The pharmacokinetic parameters of salicylic acid for the rat brain. The control group (aspirin administration 30 mg/kg only); the acupuncture group (acupuncture stimulation 15 min before aspirin administration 30 mg/kg); the electro-acupuncture group (electro-acupuncture stimulation 15 min before aspirin administration 30 mg/kg).

Parameter	Aspirin (30 mg/kg)	Aspirin (30 mg/kg) + acupuncture	Aspirin (30 mg/kg) + electro-acupuncture
*t*_1/2_ (min)	370 ± 173	345 ± 123	330 ± 184
AUC (min *μ*g/mL)	1076 ± 314	919 ± 154	810 ± 70
Cl (mL/min/kg)	21.0 ± 8.3	34.3 ± 14.0	30.7 ± 11.1
Vd (mL/kg)	8.2 ± 4.2	14.0 ± 4.9	9.1 ± 2.2

Data expressed as mean ± S.E.M. (*n* = 6). *t*_1/2_: elimination half-life, AUC: area under the concentration-time curve, Cl: clearance, Vd: apparent volume of distribution.

**Table 3:** The numeric descriptions of brain blood flow in the three groups.

Time (min)	Control group	Acupuncture group	Electro-acupuncture group
−15	942 ± 37	938 ± 47	948 ± 86
0	938 ± 42	935 ± 28	985 ± 54
5	938 ± 54	935 ± 30	988 ± 62
10	935 ± 40	932 ± 22	1022 ± 61
15	927 ± 48	957 ± 63	1020 ± 67
20	929 ± 45	952 ± 68	1026 ± 70
25	928 ± 41	956 ± 54	1025 ± 75
30	934 ± 50	958 ± 56	997 ± 63
60	934 ± 44	946 ± 61	1005 ± 115
120	934 ± 54	944 ± 67	946 ± 45
180	931 ± 41	935 ± 69	965 ± 103
230	932 ± 50	926 ± 69	963 ± 98
300	937 ± 40	925 ± 43	949 ± 78
360	936 ± 43	918 ± 49	955 ± 67

Each group contained six rats (*N* = 6).

Results were expressed as mean ± standard deviation, and the unit of blood flow is BPU.
